# 

**Published:** 1904-10-01

**Authors:** 


### The Hospital

A JOURNAL OF
THE MEDICAL SCIENCES AND HOSPITAL ADMINISTRATION.

CONDUCTED BY

SIR HENRY BURDETT, K.C.B.

JOINT EDITORS

HAROLD BURROWS, M.B., F.R.C.S.,

AND

CHARLES OLIVER HAWTHORNE, M.D., M.R.C.P.

SURGEON GENERAL'S OFFICE
DEC. 17 1906
190235

**VOLUME XXXVII.**

OCTOBER 1904 TO MARCH 1905.

London:

PUBLISHED BY THE SCIENTIFIC PRESS (LIMITED), AT THE HOSPITAL BUILDING,
28 & 29 SOUTHAMPTON STREET, STRAND, W.C.

MDCCCCV.

W1
NO69X

### INDEX TO VOL. XXXVII., 1905.

*** Articles under the general heading *Hospital Clinics* and *Medical Progress* are distinguished by the initial (C.) for *Hospital Clinics*, and by (P.) for the rest of the series. (W.) distinguishes the *Institutional Workshop* articles, and (A.) the *Annotations*.

Abdomen, The Bacteriology of Inflammatory Processes in the (C.), 189
[illegible]cess of the Lungs (P.), 9
[illegible]noids. *See* Book World, *s.v.* Wingrave
[illegible]inistration, A Suggested New Departure in Hospital (W.), 162, 178, 195
[illegible]enalin (P.), 9
[illegible]vertisements, Fraudulent (A.), 381
[illegible]rica, Anti-Malarial Measures in (A.), 419
[illegible]ir-pollution: Bacterial (P.), 124; Bacterial Test for (C.), 244
Albuminuria as a Complication of Pregnancy (C.), 103
Albumosuria, Myelopathic (C.), 331
Alcohol (P.), 209; and Commercial Efficiency (A.), 459; and Digestion (C.), 463
Amaurosis and Retro-Bulbar Neuritis (C.), 5
Amputations, Osteoplastic (C.), 25
Anaæmia, Pernicious (C.), 43, 68; Splenic (P.), 371; of Infancy (P.), 335
Anæsthetics (P.), 388, 406
Animals, Diseases of, in 1904, 292
Ankylostoma (P.), 90
Anthrax at Bermondsey, 204
Apoplexy, Traumatic (C.), 122
Appendicitis (P.), 332; Bacteriology of (C.), 189; Intestinal Obstruction following (C.), 24; Leucocytosis and (P.), 387; a Posterior Incision for Operations in (C.), 123; up to date, 400
Appendix Vermiformis. *See* Book World, *s.v.* Battle
Appointments, 18, 34, 60, 80, 98, 114, 132, 148, 166, 182, 200, 216, 238, 256, 274, 290, 378, 398, 416, 438, 456, 472
Argyrol in Eye-work (C.), 264; in Ulcerative Colitis (C.), 7
Army Medical Service: The New Head of the (A.), 241; Promotion in the (A.), 311
Arteries, How to close Wounds of the Larger (C.), 314
Arterio-sclerosis (P.), 316, 446; of the Spinal Cord (P.), 465
Arthritis: Deformans (P.), 109; Pneumococcal (C.), 444; Rheumatoid (P.), 191
Association of Scottish Medical Diplomates (A.), 459
Assouan. *See* Book World, *s.v.* Edwards.
Asylums, Private, Visits to (W.), 80
Ataxy, the Treatment of Locomotor (C.), 422

Babinski's Reflex (P.), 158
Bacteria, in the Atmosphere, 1; Notes on (P.), 26
Bacteriology (P.), 26, 123, 225, 370; and the Rheumatic Diseases (P.), 226
Banana Preparations, 319
Banti's Disease (P.), 68
Baths, Public, in the United States (W.), 340
Beef Suet, 319
Beri-Beri (C.), 153
Bilharziosis (P.), 424
Birth-Rate in New South Wales, The, 163
Birthday Honours, 118
Blood, Diseases of the (P.), 50, 68, 90, 317, 335, 350, 386; in Early Life (P.), 317; the Passage of, by the Bowel (C.), 263; in General Paralysis (P.), 91; Pressure (P.), 446
Blood and Blood Vessels, Diseases of the (P.), 301, 316, 371
Bookkeeping. *See* Book World, *s.v.* Bale
Books Reviewed—
Advertisers' A B C, 372
Allison. Health in Infancy, 194
Bale, Sons, and Danielsson. Simple Medical Year-Book or Private Medical Ledger, 286
Balfour. First Report of the Wellcome Research Laboratories at Khartoum, 228
Battle and Corner. The Surgery of the Diseases of the Appendix Vermiformis and their Complications, 194

Books Reviewed—continued
Blandy. The Holy Communion. A Possible Source of Infection, 390
Bland-Sutton and Giles. Diseases of Women, 127
Bosanquet. Serums, Vaccines, and Toxines in Treatment and Diagnosis, 160
Bradshaw. Dictionary of Bathing Places and Climatic Health Resorts, 12
British Journal of Inebriety. Vol. II., 2
Brundage. A Manual of Toxicology, 372
Burton-Fanning. The Open-air Treatment of Pulmonary Tuberculosis, 450
Carpenter. British Journal of Children's Diseases, 410
Carruthers. Urine Examination made Easy, 161
Corner. *See* Battle and
Cowen. X-rays: their Employment in Cancer and other Diseases, 212
De Santi. Malignant Disease of the Larynx, 228
Edebohls. Surgical Treatment of Bright's Disease, 228
Edwards. Notes on Assouan, 160
Force in Advertising, 410
Freeland. The Radical Cure of Corns and Bunions, 450
Fry's Royal Guide to the London Charities, 250
Gant. Auto-Biography, 390
Gibson. Nervous Affections of the Heart, 127
Giles. *See* Bland-Sutton
Gould. A Dictionary of New Medical Terms, 450
Greene. The Schott Treatment for Chronic Heart Diseases, 286
Guerville. The Crusade against Phthisis, 390
Halliburton. Biochemistry of Muscle and Nerve, 228
Hayward. Construction of Hospitals for Consumption, 212
Hutchison. Lectures on Diseases of Children, 160
Hutchinson. The Surgical Treatment of Facial Neuralgia, 390
Indian Public Health. Edited by Dr. Newell, 72
Infants' Health Society. Conditions of Infant Life and their Effect on the Nation, 390
International Clinics, Vol. III., 194
Kaminer. *See* Senator
Kapadia. The Teachings of Zoroaster, 410
Keith. Human Embryology and Morphology, 410
Kenny-Herbert. Vegetarian and Simple Diet, 250
Lake. Handbook of Diseases of the Ear, 250
Lamb. Guide to the Examination of the Throat, Nose, and Ear, 212
Lambkin. The Treatment of Syphilis, 286
Latham. The Diagnosis and Modern Treatment of Pulmonary Consumption, 450
Laws of Health, 92
Leedham-Green. The Sterilisation of the Hands, 161
Love. Diseases of the Ear, 286
Macnaughton-Jones. Manual of Diseases of Women, 286
Mummery. The After-Treatment of Operations, 161
Oxford and Cambridge Year Book, 228
Paterson. Walsham's Handbook of Surgical Pathology, 390
Pernet. Differential Diagnosis of Syphilitic and Non-Syphilitic Affections of the Skin, including Tropical Diseases, 30
Philips. Handy-Volume Atlas of London, 127
Porritt. Religion and Health: Their Mutual Relationship and Influence, 450
Practical Advertising. Mather and Crowther, 194
Pugh. The Infectivity and Management of Scarlet Fever, 410
Reynolds-Ball. Mediterranean Winter Resorts, 160
Richards. Practical Chemistry, 92

Books Reviewed—continued
Robson. Cancer and its Treatment, 410
Senator and Kaminer (Edited by). Health and Disease in Relation to Marriage and the Married State, Vol. I., 161
Shallcross. Municipal Shortcomings: the Example of Liverpool, 72
Starr. Organic Nervous Diseases, 127
Stevenson. Wounds in War: the Mechanism of their Production and their Treatment, 30
Story of an East End Hospital, 92
Street's Newspaper Directory, 1905, 410
Tebb. Premature Burial and How it may be Avoided, 372
Thomson. The Transvaal Burgher Camps, S.A., 450
Treves. The Student's Handbook of Surgical Operations, 160
Turner. Manual of Practical Medical Electricity, 92
Tyson. A Treatise on Bright's Disease and Diabetes, 372
Vincent. The Nutrition of the Infant, 161
Walker. An Introduction to Dermatology, 286
Wanhope. Excursions of an Improvident Philosopher, 92
Who's Who, 1905, 250
Who's Who Year Book, 1905, 250
Williams. Essay on Insanity, 372
Willing's Press Guide, 1905, 250
Wingrave. Adenoids: Medical Monograph Series, No. 9, 160
Year Book of the Scientific and Learned Societies of Great Britain and Ireland, 250
Bradycardia (P.), 467
Brain, Bullet extracted from, Ten weeks after Injury (P.), 283; Tumours of the (P.), 28
Brazil, Sanitation in (A.), 203
Bright's Disease and Diabetes. *See* Book World, *s.v.* Tyson; Renal Decapsulation for Chronic (P.), 227; Surgical Treatment of. *See* Book World, *s.v.* Edebohls
Brine Crystals, 335
British Medical Association (W.), 435
Burial, Premature (W.), 416, 458. *See* Book World, *s.v.* Tebb

Calculus: Prostatic, Fracture of Spine Complicated by Large (P.), 28[illegible]; Renal, Gigantic (P.), 249
Canal, Alimentary, Bacteria in Inflammatory Diseases of the (P.), 227
Cancer: (C.), 244; Larynx. *See* Book World, *s.v.* di Santi; The Medicinal Treatment of (C.), 189; The Treatment of, 183 (C.), 188; Research (P.), 91, 107, (C.) 282. *See* Book World, *s.v.* Cowen and Robson
Carcinoma: The Causation of (C.), 331; The Etiology of (C.), 423; of the Pancreas (P.), 141; of the Prostate, Primary (P.), 71; of the Bulbous Urethra (P.) 71
Carpal Bones, Fractures and Dislocations of the (C.), 25
Catarrh, Spring (P.), 11
Cavernous Sinus, Thrombosis of the (P.), 8
Cerebral Infection, On the Path and Nature of (P.), 177
Cerebral Surgery, Progress in (P.), 282
Cerebral and Nerve Surgery, Progress in (P.), 27, 177
Certificate, The Claims of the (A.), 259
Chamberlain, Mr., and the Medical Profession (A.) 293
Characters, Physical, and Morbid Proclivities (C.), 245
Chemist-Opticians (A.), 135
Chemistry. *See* Book World, *s.v.* Richards

190235

Chest: The Shape of the (P.), 449; Injuries to the Wall (P.), 10
Chiasma, Tumours involving the Optic (P.), 232
Children, Diseases of. *See* Book World, *s.v.* Hutchison; Feverishness in (C.), 297; Imbecile (C.), 367; Separate Courts of Justice for (W.), 130; Sudden and Unexpected Death in (C.), 223
Chloroform, 11, (P.), 388; Ethics of Administration, 98; Poisoning, Delayed (P.), 406
Chlorosis (C.), 43
Cholecystectomy (P.), 351
Cholecystitis, Bacteriology of (C.), 189
Chorea (P.) 385
Chorion-Epithelioma (C.), 224
Choroidal Inflammation with Loss of Vision caused by Excessive Use of the Eyes for a Short Period (P.), 11
Circulatory System, Diseases of the (P.), 447, 467
Circumcision as an Aid to Identity (A.), 259
Cirrhosis, Hepatic (P.), 351
Clergy and Medical Fees (A.), 419
Climates, The Therapeutic Value of Relaxing, (C.), 421
Coal-Gas Poisoning (P.), 427
Coagulation (P.), 447
Cod-liver Oil, 335
Colitis, Argyrol in Ulcerative (C.), 7; Mucomembranous (P.), 157
Collargulum, 267
Colon, Atony and Dilatation of the (P.), 158; Diseases of the (P.), 157; Syphonage in the (P.), 158; Ulcerative Affections of the (P.), 157
Coma, Diabetic (P.), 193
Conferences: International Tuberculosis, 12; International Congress of Medicine, Lisbon, 1906 (W.), 358; International Sanitary Convention, Paris (A.), 219
Constipation and Diarrhœa (P.), 158; Olive-oil Injections in (C.), 68
Construction Notes (W.), 59, 198, 274, 323, 376
Consultations between Medical Witnesses (A.), 345
Consumption. *See* Book World, *s.v.* Latham; Hospitals for, Construction of. *See* Book World, *s.v.* Hayward
Convulsions in Infancy and Childhood (C.), 366
Co-operation, Want of (W.), 373, 392, 468
Corns and Bunions. *See* Book World, *s.v.* Freeland
Cottages, Cheap (A.), 241
Cranial Depression, Operative Treatment of (P.), 177
Criminals, Juvenile (A.), 135
Currents, High Frequency (P.), 300
Curriculum, The Five Years' (A.), 185
Cystic Disease (P.), 449
Cysts, Lutein (C.), 224; Mesenteric (P.), 248

Death-Roll, The Industrial (A.), 401
Decapsulation, Renal, for Chronic Bright's Disease (P.), 227
Decay, Mental Disorders of (C.), 424
Decimal Association, The, 324
Dementia, Katatonic (P.), 285
Dermatitis, Blastomycetic (P.), 49
Dermatology, Progress in (P.), 48
Diabetes (P.), 193, 208, 224; and Bright's Disease. *See* Book World, *s.v.* Tyson; Diet and Drugs in (C.), 172; Mellitus (C.), 42; The Pancreas and (P.), 126; Diagnosis, a Case for (C.), 348; of some Kidney Diseases, A Difficulty (P.), 449
Diarrhœa, Constipation and (P.), 158
Digestion, Alcohol and (C.), 463
Dilatation, Tetany and Gastric (P.), 266
Diphtheria, Faucial, Early Sequelæ of, and their Treatment (C.), 405
Discussions, The Multiplication of (A.), 441
Disorders, Functional Mental (C.), 347
Distress, The Relief of, 81
Doctors in Ancient Ireland, 52
Dress, Doctors and (A.), 37
Droitwich as a Health Resort (W.), 272
Drugs, the Purity of (A.), 441
Drunkard, the Habitual (A.), 293
Dundee "Social Union," The, 440
Duodenal Ulcer and its Treatment (C.), 172
Dustbin in "Progressive" Putney, The (W.), 253
Dymal, 467
Dysentery (P.), 26
Dyspepsia in Children, Mucous Disease or Chronic Intestinal (C.), 384

Eclampsia (P.), 210; Puerperal (C.), 137
Economy in Scottish Hospitals (W.), 452
Editor's Letter Box—
Aerated Waters, 360
Antivivisection Statistics, 17
Decimal Association, 324
Dried Milk, 290
East London Medical Society and Hospital Abuse, 113
Ethics of Chloroform Administration, 98
Great Northern Central Hospital, 455
Editor's Letter-Box—continued
High-frequency Currents and the Treatment of Sciatica, 131
Hospital Attendants and Male Servants, 290 and 308
Hospital Expenditure and Efficiency, 472
Hospital Expenditure and its Reduction, 303
Italian Hospital, 360
London Orphan Asylum, Watford, 308
Medical Practitioners and Hospital Abuse, 148
Movable Kidney, 324
National Hospital for Paralysed and Epileptic, 216
Nursing at Woodilee Asylum, 60
Premature Burial, 416
Queen's Jubilee Hospital, 397
Royal Infirmary, Glasgow, 455
Royal Orthopædic Hospital, 324
St. John's Hospital for Diseases of the Skin, 34
University College Hospital, 199
Education of Medical Students, The Preliminary, 184; Medical, in London (C.), 383; Physical (A.), 151; Physical, Sir James Crichton Browne on, 35
Effusion, Pleural (P.), 10
Embolism and Thrombosis of the Vessels of the Mesentery (P.), 426
Embryology and Morphology, Human. *See* Book World, *s.v.* Keith
Emergency Receiving House, or First Aid Station (A.), 185
Empyema, Interlobar (C.), 366; Iodine Bath Treatment of (C.), 421
Endocarditis, Infective (P.), 467
Enemata, Vomiting of (O.), 7
English of an Elizabethan Physician, The, 73
Entente Cordiale, L', 20
Enteroptosis and Neurasthenia (C.), 384
Epigastric Pain in Acute Rheumatism in Children (P.), 109
Epilepsy (P.), 159; Influence of Dentition in the Etiology of (C.), 263; and Insanity (P.), 464; the Surgical Treatment of (P.), 177
Epiphysis, Separation of the (C.), 105
Epithelioma of the Tongue in Women (C.), 106
Erythema Infectiosum (P.), 48
Ethyl Chloride (P.), 407
Eucaine Lactate (C.), 47
Evacuant, Strychnine as an (C.), 8
Examiners and Inspectors (A.), 203
Excretion, On (C.), 295
Exophthalmos, The Mechanism of (C.), 139
Expenditure, Hospital, and its Reduction (W.), 287, 305, 308; of the London Hospitals, a Ten Years' Review of the (W.), 338; with Efficiency, the Control of Hospital, 417, 420; (W.), 430, 451, 452
Expert's Opinions, An (A.), 63
Eye-strain as a Cause of Headache and other Neuroses (P.), 10

Feeble-minded, Guildhall Conference (W.), 96; Self-supporting Colonies for the (A.), 219
Feeding of Infants, Lectures on the (C.), 221, (C.), 261, (C.), 279
Femur, Fracture of the Neck of the (C.), 105, 143
Fever, Enteric, Therapeutic Fasting in (C), 207; Typhoid, Intestinal Perforation in (P.), 426; Typhoid, Perforation of the Intestine in (C.) 444; Typhoid, at Lincoln, 399; Public Prayer and Typhoid (A.), 363; Scarlet, The Diagnosis of (C.), 349; Scarlet. *See* Book World, *s.v.* Pugh; Yellow, and Mosquitoes, 8, 36
Feverishness in Children (C.), 297
File System, Stolzenberg (W.), 256
Finsen, Professor, 2
Fire Protection at the Portsmouth Hospital (W.), 33
Flats, The Multiplication of Unhealthy (A.), 63
Flies, Infection by (P.), 466
Foci in Bone, The Origin of Inflammatory (P.), 143
Fog and Smoke, 258
Food and Feeding, 219
Foods, Artificial (C.), 279
Foot-warmer, Bed and, 378
Formosa, Japanese in (A.), 3
Fracture, Compound (C.), 119
Fractures of the Skull (P.), 28

Ganglion, Gasserian, Surgery of the (P.), 27
Gangrene, Emphysematous (P.), 124
Garlic Treatment (P.), 51
Gastro-jejunostomy (C.), 365
Gastro-enterostomy (P.), 246
General Medical Council, The, 167
Genito-Urinary Diseases (P.), 71, 106
Genito-Urinary Surgery (P.), 227, 249, 368, 389
Glances at the Hospitals (W.), 15, 33, 57, 78, 97, 131, 165, 182, 215, 255, 274, 290, 397, 415
Glands: Diseases of Certain (P.), 407; Lymphatic, Affections of the (P.), 351; Thyroid, Diseases of the (P.), 407; the Supra-renal (P.), 408
Glycogen Reaction in Blood, The (P.), 387
Glycosuria, Experimental (P.), 193
Gout: (P.), 192; Rheumatism and (P.), 108, 191
"Goutte de Lait," The (W.), 13
Guardians' Subscriptions to Voluntary Hospitals (W.), 322
Guild of St. Luke's (W.), 97

Hæmorrhage, Treatment of Accidental (P.), 210; in Pregnancy, Accidental (C.), 245
Hands, the Sterilisation of the. *See* Book World, *s.v.* Leedham-Green
Headache and other Nuroses, Eyestrain as a Cause of (P.), 10
Health, Assurance (A.), 311; Laws of. *See* Book World, *s.v.* Laws: Problems, Local Authorities and (A.), 277; Resorts *see* Book World, *s.v.* Bradshaw
Heart. *See* Book World, *s.v* Gibson; and its Examination, the (C.), 313; Diseases. *See* Book World, *s.v.* Greene; Massage of the (P.), 407
Hemiplegia in Cerebral Tumour (P.), 464; Uræmic (P.), 159
Hernia, Cerebri and Septic Meningitis, Recovery from (P.), 177; in Children (P.), 264; Radical Cure of (P.), 265; Strangulated (P.), 283; Progress of Surgery of (P.), 283; Progress of Surgery in (P.), 264; Umbilical (C.), 140; of the Uterus through the Inguinal Canal (P.), 285; Ventral, the Prevention of, as a Sequel to Abdominal Section (P.), 284
Homes, Convalescent (W.), 437; Regimental (W.), 14
Honours to Members of the Medical Profession (C.), 245
Hospital Abuse and Medical Practitioners, 113, 116, 148
Hospital Attendants and Male Servants, 290, 308
Hospital Work, The Engineering Side of (W.), 321, 339, 340, 375, 411, 428, 453, 469
Hospital, Jewish Opposition to a Jewish (A.), 151
Hospital Officers' Association, The (W.), 94
Hospital Library and Charities Bureau, The (W.), 18, 416, 429, 472
"Hospital Penny Fund," The, 4, 38 (W.), 53 (W.), 74 (W.), 95 (W.), 129 (W.), 145
Hospital Saturday Fund (W.), 323
Hospital Sunday Fund (W.), 163 (W.), 288 (W.), 235; The London Commercial Travellers and the (W.), 252; The Constitution of the (W.), 251 (W.), 270; and the Special Hospitals (W.), 288
Hospitals and the Funds, Small (W.), 144, 179, 196, 214
Hospital and Institutions, London—
Acton:
Cottage Hospital (W.), 59
Isolation Hospital (W.), 323
After-Care Association (W.), 414
Bloomsbury, Queen Square, the National Hospital for the Paralysed and Epileptic (W.), 57, (W. 147)
British Home for Incurables, 118, 186, 364
Brompton:
Cancer Hospital, 118 (W.), 185; 346, 364
Hospital for Consumption, 118, 364
Brondesbury, St Monica's Home Hospital for Sick Children (W.), 472
Charing Cross Hospital, 118, 260, 364, 396, New Out-Patient Department (W.), 78; Medical School (W.), 180
Chelsea Hospital for Women, 118 (W.), 256, 364, 382
Chelsea Lister Institute, 152
City of London Hospital for Diseases of the Chest, 364
City of London Lying-in Hospital (W.), 397
City of London Truss Society (W.), 238, (W.), 359
City Road Royal Chest Hospital (W.), 470
Dartford, City of London Asylum (W.), 79
Earlswood Asylum for Idiots, 364
East London Hospital for Children, 364, 382. *See* Book World, *s.v.* Story
Evelina Hospital, 364
Female Lock Hospitals, 364
French Hospital (W.), 471
Fulham Cancer Hospital (W.), 413
German Hospital (W.), 341
Great Northern Central Hospital, 455
Great Ormond Street Hospital for Sick Children (W.), 97, 152, 364
Greenwich: Miller and Seamen's Hospital, 186
Guy's Hospital, 364
Hospital for Diseases of the Throat (W.), 454
Ilford: Proposed Emergency Hospital (W.), 214
Italian Hospital (W.), 342, 360
Kensington Dispensary, 118
King's College Hospital, 22, 118, 260, 364, (W.) 412
London County Asylums, Pathological Research in, 100
London Fever Hospital, 118, 364
London Hospital, 118, 185, (W.) 215, 364; Opening of New Jewish Wards (W.), 147; Out-patients, 64

Hospitals and Institutions, London—continued
London School of Tropical Diseases, 220
Metropolitan Hospital, 364
Middle-ex County Asylum, Wandsworth (W.), 17
Middlesex Hospital, 118 (W.), 181, 260, 364
National Hospital for Diseases of the Throat (W.), 455
National Hospital for the Paralysed and Epileptic, 364
National Orthopædic Hospital (W.), 414
New Hospital for Women (W.), 271
North-Eastern Hospital for Children (W.), 164
North-West London Hospital (W.), 198
Queen Charlotte's Hospital, 186 (W.), 437
Queen's Jubilee Hospital (W.), 392, 397
Richmond, Surrey, Royal Hospital (W.), 255
Royal Dental Hospital of London, 152 (W.), 470
Royal Eye Hospital (W.), 471
Royal Free Hospital, 118
Royal Hospital for Incurables, Putney Heath, 118 (W.), 182, 346, 364, 460
Royal London Ophthalmic Hospital, 364 (W.), 414
Royal Orthopædic Hospital, 324
Royal Waterloo Hospital for Children and Women, 186 (W.), 307
Royal Westminster Ophthalmic Hospital (W.), 454
St. Bartholomew's Hospital, 38, 112 (W.), 337; How It can be Saved, 99; The Opportunity of, 61
St. George's Hospital, 118 (W.), 131, 242, 260, 364
St. Mark's Hospital (W.), 377
St. Mary's Hospital, 118, 364, 460
Salford Sick Poor Nursing Institute, 382
Samaritan Free Hospital, 364 (W.), 471
Seamen's Hospital Society (W.), 307, 364 (W.), 413
Thames Ditton Cottage Hospital (W.), 274
Tottenham Hospital (W.), 129, (W.), 413
University College Hospital, 118, 199, 364 (W.), 470
Victoria Hospital for Children, 118, 364
West End Hospital (W.), 455
West London Hospital, 260, 460
Westminster General Dispensary (W.), 323
Westminster Hospital, 118; (W.), 198, 364
Walthamstow, Children's Hospital (W.), 255
Watford, London Orphan Asylum, 308
York Road, General Lying-In Hospital (W.), 341
Provincial—
Aberdeen:
Maternity Hospital, 402
Royal Asylum (W.), 17
Alnwick, New Hospital, 402
Aston Union Workhouse Infirmary (W.), 198
Axbridge Workhouse Infirmary (W.), 76
Ayr County Hospital (W.), 97
Barnstaple, North Devon Infirmary, 294
Bedford County Hospital (W.), 397
Belfast Maternity Hospital (W.), 199
Bexhill Convalescent Home, 364
Birkenhead Borough Hospital (W.), 415
Birmingham:
Free Hospital for Sick Children (W.), 33
General Hospital (W.), 415
Blackburn Infirmary (W.), 79
Blackpool Victoria Hospital (W.), 16
Blencathra, Cumberland, Consumption Hospital, 22
Brighton Hospital, the (W.), 32
Bristol:
Dispensary, 346
Eye Hospital, 346
General Hospital (W.), 165, 346
Royal Infirmary, 346
Cambridge, Addenbrooke's Hospital (W.), 128, 294
Cambridge County Asylum (W.), 17
Campbelltown Cottage Hospital, 136
Cardiff Infirmary (W.), 59, 278, 382
Chalfont St. Peter's Epileptic Colony (W.), 59
Chester Infirmary, 420
Chesterfield Isolation Hospital (W.), 274
Cork, North, Charitable Infirmary, 170
Coventry and Warwickshire Hospitals (W.), 57
Crewkerne Hospital (W.), 198
Cumberland and Westmorland Asylum (W.), 164
Derby Borough Asylum (W.), 79
Doncaster and Mexboro' Infectious Hospital (W.), 55
Dublin:
Hervey Lewis Memorial Hospital, 242
New Orthopædic Hospital, 402
Dumbarton Cottage Hospital (W.), 272
Dundee:
Royal Infirmary (W.), 274
Royal Victoria Hospital (W.), 193
Dunfermline Cottage Hospital, 152, 220
Durham County Hospital (W.), 33
Durham, North, Eye Infirmary (W.), 59
Eastbourne, All Saints' Convalescent Home, 364, 460
Edinburgh:
Royal Infirmary (W.), 165, 220
Royal Victoria Hospital, 242
Essex and Colchester Hospital (W.), 131

Hospitals and Institutions, Provincial—continued
Exeter City Workhouse (W.), 199
Folkestone Victoria Hospital (W.), 78
Fullerton Accident Hospital, 196
Glamorgan County Asylum (W.), 273
Glasgow:
Eastern District Hospital, 4
General Hospital, 4
Maternity Hospital (W.), 196
Royal Infirmary (W.), 15, 136, 220, 455
Victoria Infirmary, 136
Western District Hospital, 4
Western Infirmary, 136, 220, (W.) 303
Grange-over-Sands Convalescent Home, 170
Greenock Hospital and Infirmary (W), 78
Halifax Royal Infirmary, 460
Hambledon Proposed Isolation Hospital (W.), 59
Hants, Royal West (W.), 215
Hereford and Westley Rural District Isolation Hospital, 22
Herefordshire General Hospital (W.), 255
Horwich Infectious Diseases Hospital, 460
Hull:
Dispensary, 136
Fishermen's Hospital (W.), 97
Royal Infirmary, 136, 152
Victoria Hospital for Children, 136
Ipswich Hospital (W.), 59
Isle of Man, Noble's Hospital, 220
Isle of Wight, Royal Hospital, 460
Lanark and Govan, Kirklands Asylum (W.), 80
Lancaster Royal Infirmary (W.), 33
Leeds:
Hospital for Women and Children (W.), 16
Infirmary, 402
Killingsbeck Small-pox Hospital, 22
Seacroft Hospital, 22
Leicester and Rutland Asylum (W.), 58
Limerick District Asylum (W.), 58
Limpley Stoke, Bath, Winsley Sanatorium for Consumptives (W.), 320
Liverpool:
Bluecoat Hospital (W.), 323
Hospital for Consumption, 278
Royal Infirmary (W.), 323, 442
Royal Northern Hospital (W.), 323
Royal Southern Hospital (W.), 323, 442
School for the Blind (W.), 323
School of Tropical Medicine, 4
School of Tropical Diseases, 136
Stanley Hospital, 102
University Medical School, 136
Lowestoft Hospital (W.), 273
Macclesfield General Infirmary, 4; (W.), 16
Manchester Hospital for Skin Diseases (W.), 31
Mansfield Hospital, 152
Margate, Royal Sea-Bathing Hospital, 364
Monmouthshire Asylum (W.), 198
Montgomeryshire Infirmary (W.), 290
Morton Isolation Hospital, 278
Montrose Royal Asylum (W.), 164
Mullingar District Asylum (W.), 34
Newton Abbot Hospital (W.), 16
Northwood, Mount Vernon Hospital for Consumption, 4
Nottingham and Notts Sanatorium, 170
Norfolk and Norwich Hospital, 64
Nuneaton, Proposed Small-pox Hospital, 460
Oldham Infirmary (W.), 78
Perth:
Murray's Royal Asylum, 22
Royal Asylum (W.), 359
Portsmouth Borough Asylum (W.), 273
Plymouth Borough Asylum (W.), 165
South Devon Hospital (W.), 16
Portsmouth Royal Hospital, 4 (W.), 53
Rotherham Hospital (W.), 97
St. Helen's Hospital, 278
St. Leonards, Eversfield Hospital, 84
Salisbury Infirmary, 33
Salop and Montgomery Asylum (W.), 415
Scarborough Hospital (W.), 182
Sheffield Royal Hospital, 84
Shrewsbury, The Salop Infirmary (W.), 144
South Shields Union Infirmary, Nurses' Home (W.), 377
Southampton Hospital (W.), 58
Stamford and Rutland Infirmary (W.), 255
Stroud Infectious Diseases Hospital, Cashes Green (W.), 274
Sunderland:
Borough Asylum (W.), 79
Eye Infirmary, 260
Infirmary (W.), 59
Royal Infirmary, 260
Surrey:
Brookwood County Asylum (W.), 254
Royal County Hospital, 242
Taunton and Somerset Hospital (W.), 93
Tredegar Cottage Hospital, 84
Ulverstone Cottage Hospital (W.), 59
Wakefield and Clayton Hospital (W.), 273
Walsall Hospital (W.), 165
Wanstead, Infant Orphan Asylum (W.), 254
West of Scotland:
Association for the Relief of Incurables, 136
Convalescent Home, 136

Hospitals and Institutions, Provincial—Continued
Windsor Royal Dispensary and Infirmary, 64
Wolverhampton Hospital for Women (W.), 93
Woodilee Asylum, 60
Worcester General Infirmary (W.), 397
Colonial and Foreign—
Adelaide, Children's Hospital (W.), 58
British Columbia Hospital for the Insane (W.), 95
Cannes, British Hospital at Sunny Bank (W.), 77
Rome, New Hospital for Strangers (W.), 253
Sydney, Royal Prince Alfred Hospital (W.), 59
Hospitals and Medical Schools (W.), 111; (W.), 130, 394
Hospitals, Special, The Work of (A.), 401
Hospitals of London, The Resources and Requirements of the Voluntary (W.), 354
Hydatid Disease (P.), 424
Hydronephrosis from Papilloma of the Renal Pelvis (P.), 107
Hygiene, Institute of (A.), 83
Hyoscine Hydrobromide, (P.), 407
Hysteria and Neurasthenia (P.), 465; and Ocular Paralysis (C.), 121

Immunisation, a Curiosity of (C.), 462
"In Corpore Sano" (A.), 381
Incision and Suture of the Abdominal Wall (P.), 248
India, The Health of (W.), 253
Industrialism and Infant Mortality (A.), 345
Inebriate, Control of the (W.), 213
Inebriates, Women (W.), 117
Inebriety. *See* Book World, *s.v.* British Journal of Infancy, Health in. *See* Book World, *s.v.* Allison
Infant: at Birth, The (C.), 423; Nutrition. *See* Book-World, *s.v.* Vincent; Artificial Feeding of, Excess of Fat in the (C.), 315; The Overlaying of, 240; Lectures on the Feeding of (C.), 22 (C.), 251 (C.), 279
Infection by Flies, (P.), 466; Puerperal (P.), 210
Inhalers (P.), 388; New Regulating, 29
Inoculation, Anti-Typhoid, 133; and Plague in India (A.), 459
Insane from Year and Year, Story of the (W.), 17, 34, 58, 79, 164, 198, 254, 273, 359, 415
Insane. *See* Hospitals, &c., *s.v.* British Columbia
Insanity, with Permanent Partial Dementia, Arrest of the Progress of (P.), 298; Epilepsy (P.), 464; in the Malay and Egyptian Races (P.), 299; Manic-Depressive (P.), 141, 156; in New Zealand, 260; the Treatment of Incipient (C.), 422; Treatment of, by Hypnotics (P.), 207; Frontal Lobe Tumours and (P.), 299. *See* Book World, *s.v.* Williams
Intestine, Exclusion of the (P.), 425
Intestines, Surgery of the (P.), 425
Intubation, Tracheotomy and (C.), 298
Invalid Children's Aid Association (W.), 77

Japanese in Formosa (A.), 3
Jaundice, Prolonged (C.), 86
Joint Disease of Childhood: An Obscure Multiple (C.), 403

Kernig's Sign (C.), 24
Kidney Extract (P.), 409
Kidneys, Diseases of the (P.), 318, 333, 408, 424, 449; Unsuspected Lesions in Movable (P.), 227; Movable (C.), 280, 324
King Edward's Hospital Fund for London (A.) 151, (W.) 163, (W.) 229, (W.) 269; and the Hospitals, 217
Knee-jerk in Pneumonia, the (C.), 46
Kollman, Five-glass Test in Urethritis (P.), 106

Labours: Notes of 100 Consecutive Successful Operations from over 2,000 Aggregate (C.), 329
Laminectomy for Injuries of the Spinal Cord (P.), 27
Larynx, Cancer of the. *See* Book World, *s.v.* de Santi
League of Mercy (W.), 57, 237, 251, 270; Grants in 1904, 312
Lectures, The Futility of (A.), 21
Leg Bones, the Ambulatory Treatment of Fractures of the (P.), 143
Leishman-Donovan Body, the (P.), 123; Cultivation of Trypanosoma from the (C.), 281
Lesions in Movable Kidneys, Unsuspected (P.), 227; of the Cervical Sympathetic Nerves (C), 365; Pancreatic (P.), 193; Spinal (P.), 208
Leucocytes, Eosinophile (P.), 387
Leucocytosis and Appendicitis (P.), 387
Leukæmia (P.), 69; in Childhood (P.), 350; and Sarcomatosis (P.), 337; Acute Lymphatic (C.), 406
Liberal Views and the Medical Profession, 52
Lincoln, Typhoid Fever at, 399
Lithotrity for Large Vesical Calculi (P.), 368
Liver, Abscess (P.), 351; Cirrhosis of the (C.), 23
London, The Health of, 291

London School of Economics and Political Science (A.), 83
Looking Back, 239
Lumbago, Notes on Sciatica and (C.), 347
Lunacy, Forty-sixth Annual Report of the Commissioners in, for Scotland (W.), 15
Lungs, Abscess of the (P.), 9
Lymphocyte, Rôle of the (P.), 386
Lysol, 409

Maggi's Essence, 409
Magnesia, Milk of, 319
Maltine with Creosote, 409
Man *versus* the Microbe (C.), 405
Marriage. *See* Book World, *s.v.* Senator; and Tuberculosis (A.), 3
Measles in London, 325
Measures, Medicine (A.), 277
Medicine: (C.), 245; State (C.), 243; Tropical (C.), 243
Medico-Legal Society, The (A.), 63
Meetings, Hospital (W.), 181, 215, 238, 307, 341, 359, 377, 396, 412, 437, 454, 470
Megalerythema Epidemicum (P.), 48
Melancholia, Intermittent or Periodic (P.), 156
Meningitis (C.), 41; Cerebro Spinal (P.), 174; Septic, and Hernia Cerebri, Recovery from (P.), 177; Tuberculous, in Adults (P.), 159
Mesentery, Embolism and Thrombosis of the Vessels of the (P.), 426
Methylene Blue, Excretion of (P.), 409
Micro-organisms in Cæcal Digestion, Action of (P.), 124
Micturition, The Treatment of Involuntary (P.), 369
Migraine (P.), 8; and Ocular Paralysis (C.), 122
Milk: Bacterial Contamination of, 82; Dried, 290; Powders (W.), 255; The Sale of Infectious (A.), 117; Supply (C.), 244; Supply, a Model (C.), 187; Supply, a Municipal (A.), 363. *See* "Goutte de Lait"
Miners' Phthisis (P.), 227
Mole, Vesicular (C.), 224
Morphology and Embryology, Human. *See* Book World, *s.v.* Keith
Mortality, Infant, and Industrialism (A.), 345
Mosquitoes and Yellow Fever, 36
Motation, Apoplectic (C.), 88
Motions, Reversals of Habitual (P.), 9
Muscle and Nerve, Biochemistry of Muscle and Nerve. *See* Book World, *s.v.* Halliburton
Myasthenia Gravis (C.), 464
Myositis Ossificans Traumatica (P.), 143

Names and Theories (C.), 385
National Hospital for the Paralysed and Epileptic, 457
Necrosis of the Pancreas (P.), 141
Nephritis, Some Rarer Forms of (C.), 7; Treatment of (P.), 409; in the Young (P.), 333
Nerve Defects, Bridging (C.), 188
Nerve, Fifth, Surgery of the (P.), 127
Nerve Grafting, Infantile Paralysis Cured by (P.), 282
Nerve-trunks, Perforating Wounds of (P.), 283
Nerves, Cervical Sympathetic, Lesions of the (C.), 365
Nerves and Electrical Treatment, Diseases of (P.), 300
Nervous Diseases, Organic. *See* Book World, *s.v.* Starr
Nervous System, Progress in Diseases of the (P.), 60
Neuralgia, Facial. *See* Book World, *s.v.* Hutchinson
Neuralgias, Classification of, by their Reaction to Cocaine Injections (P.), 283
Neurasthenia, Enteroptosis and (C.), 384; Hysteria and (P.), 465
Neuritis (P.), 208: Amaurosis and Retro-Bulbar (C.), 5; Retro-ocular (P.), 175
Neurology, Progress in (P.), 8, 124, 158, 174, 464
New Appliances and Things Medical, 11, 29, 52, 211, 267, 319, 335, 353, 371, 467
Nodules, Subcutaneous Fibrous (C.), 66
Noises, the Suppression of (A.), 37
Nose. *See* Book World, *s.v.* Lamb
Notes and News, 4, 22, 38, 64, 84, 102, 118, 136, 152, 170, 186, 204, 220, 242, 260, 278, 294, 312, 328, 346, 364, 382, 402, 420, 442, 460
Notification of Diseases, 19
Nutrition (P.), 190

Obituary: Braidwood, Dr. Murray, 278; Cross, G. A., 112; Holden, Luther, 364; Poore, George Vivian, 170; Roose, Robson, 364; Russell, Dr. J. B. (A.), 101; Vintras, Dr. Achille, 136
Obstetrics (P.), 210
Obstruction, Intestinal (P.), 425; following Appendicitis (C.), 24
Ocular Paralyses (C.), 120; and Intra-cranial Disease (C.), 120
Œdema of the Glottis (C.), 331
Olive Oil Injections in Constipation (C.), 68
Open-air Treatment (P.), 51
Operations, The After Treatment of. *See* Book World, *s.v.* Mummery
Operations in Private Practice, Surgical (C.), 85, 119, 154, 171, 205
Operations, Surgical. *See* Book World, *s.v.* Treves: The Preparation of Rooms and Instruments in (C.), 171
Ophthalmology, Progress in (P.), 10
Opthalmoscope in Medical Practice, The (C.), 39
"Optology, Procedure in" (A.), 169
Organo-Therapy in Ancient Times, 268
Orthoform in the Diagnosis of Gastric Ulcer (C.), 88
Osteitis Deformans (P.), 143
Osteomyelitis of the Spine, Acute (P.), 28
Osteoarthropathy, Hypertrophic (P.), 192
Overlaying of Infants, The, 240
Oysters and Bacilli (A.), 401

Pancreas, Carcinoma of the (P.), 141; Diseases of the (P.), 126, 140; Lessons of the (P.), 193; Necrosis of the (P.), 141; Sarcoma of the (P.), 141
Pancreatitis, Acute and Chronic (P.), 140
Paralysis, Some Ocular (C.), 120
Paralysis, General, The Blood in (P.), 91; Infantile, Cured by Nerve-grafting (P.), 282; Musculo-Spiral, Transplantation of Tendon for (P.), 177; of the Palmar Branch of the Ulnar (P.), 300; Post-diphtheritic Chronic Bulbar (P.), 176
Papilloma of the Renal Pelvis, Hydronephrosis from (P.), 107
Paranoia (P.), 176
Paraplegia, Senile (P.), 9
Parasites, Intestinal, the Symptoms Caused by (C.), 313
Parotitis and Gastric Ulcer (C.), 207
Patella, Treatment of Fractured (P.), 143
Pathology, Surgical. *See* Book World, *s.v.* Paterson, 390
Patients and the Medical Profession, Free Hospital (W.), 391
Percussion Dulness, on Shifting (C.), 443
Perforation of the Intestines in Typhoid Fever (P.), 426, (C.), 444
Perimeningitis, Acute Suppurative (P.), 28
Peritoneum, Surgery of the (P.), 248
Peritonitis, the Causation of (C.), 403; Diffuse (P.), 248; Pneumococcus (C.), 208
Perrier Water, 335
Pharmacists and the Pharmacopœia (A.), 363
Pharmacopœias, Hospital, 257
Philosophy and Medicine, 110
Phimosis, Acquired (P.), 369
Phthisis, Fibroid (P.), 427; Miners' (P.), 227; Racial (P.), 466; The Use of Raw Meat in the Treatment of (C.), 297. *See* Book World, *s.v.* Guerville
Pituitary Body, Tumour of the (P.), 9
Plague and Inoculation in India (A.), 459
Pleural Cavity, Surgical Interference with the (C.), 154
Pleural Effusion (P.), 10
Plexus, Brachial (P.), 300
Pneumonia, The Knee-jerks in (C.), 46; The Treatment of (C.), 315.
Poisons, The Dispensing of (A.), 327
Poor of London, The Houseless, 439
Poor-Law Administration, Notes on (W.), 289
Posology, An Orthodox (A.), 203
Practical Departments (W.), 17, 199, 256, 378, 416
Pregnancy: Albuminuria as a Complication of (C.), 103; Accidental Hæmorrhage in (C.), 245; Pyelitis Complicating (P.), 211.
Preparations, Proprietary, and the Medical Profession, 276
Prescription, The Signature of the (A.), 345
Prolapse, Rectal, The Treatment of, by the Submucous Injection of Paraffin (C.), 6 (P.), 389
Prostate, Primary Carcinoma of the (P.), 71
Prostatectomy: Perineal (P.), 249; Suprapubic (P.), 249
Pruritus Ani, Treatment of (C.), 206
Psoas Abscess (C.), 119
Psychiatry (P.), 141, 156, 176, 267, 285, 295
Pyelitis Complicating Pregnancy (P.), 211
Pylorus, Congenital Hypertrophic Stenosis of the (C.), 44 (C.), 463

Quacks, A Catalogue of, 344
Questions, Hospital, which Press (W.), 373, 393, 468

Rabies (P.), 26
Rats on Shipboard, 201
Records and Medical Research, Hospital, 200
Rectal Prolapse, Treatment of, by the Submucous injection of Paraffin (C.), 6
Red Cross Society, A, 149
Red Cross Society, The Russian (A), 101
Renal Disease (P.), 42
Representation of the Universities of Glasgow and Aberdeen, The Parliamentary (A.), 117
Resorts, Winter. *See* Book World, *s.v.* Edwards, Reynolds Ball
Respiratory Diseases (P.), 448, 465
Respiratory Organs, Diseases of the (P.), 426
Respiratory System, Diseases of the (P.), 9, 28, 51
Respiratory Tract, Diseases of the, Treatment of, by Superheated Compressed Air and Comminuted Medicated Vapours (C.), 138
Revaccination, The Government and (A.), 441
Reversals of Habitual Motions (P.), 9
Revivals: The Medical Aspects of Religious Revivals, 343
Rheumatism: Acute (P.), 108, 370; Epigastric Pain in, in Children (P.), 109; and Gout (P.), 108, 191
Rhizomélique, Spondylose (P.), 192
Rigor Mortis in Still-born Children (C.), 67
Royal Colleges of Physicians and Surgeons and their Members, The (A.), 219

Salpingitis in Children, Gonorrhœal (C.), 46
Salt, Worcestershire, 267
Sanitary Office, The Tenure of, 380
Sanitation: in Brazil (A.), 203; in the Field, 362
Sarcoma of the Pancreas (P.), 141
Sarcomatosis, Leukæmia and (P.), 387
Schistosoma Cattoi: a new Blood Fluke of Man (C.), 281
School Hygiene, 268, 275, 361
School and Home, 309
Schools: Industrial, the Work of the (A.), 101; Medical Inspection of, 326
Sciatica: The Treatment of (C.), 67; High-frequency Currents and the Treatment of, 131; Lumbago, Notes on (C.), 347
Sclerosis: Arterial (P.), 301; Disseminated (C.), 43; Multiple (P.), 124; Tabes Dorsalis and Disseminated (C.), 120
Seborrhœa (P.), 48
Secretaries, Honorary, the Authority of (W.), 359
Senile Paraplegia (P.), 9
Sensation, Tactile (P.), 464
Septicæmia (C.), 445
Serum: Antistaphylococcic (P.), 49; Antistreptococcic (C.), 263; Treatment (P.), 28
Serums. *See* Book World, *s.v.* Bosanquet
Sewage, The Royal Commission on (A.), 21
Shelters and Lodging Houses, 115
Ships, Japanese Red Cross Society, 242
Shock, Electric, Death from (P.), 300
Shoulder-joint, Infantile Paralysis affecting the (P.), 143
Skin. *See* Book World, *s.v.* Pernet
Skull, Fractures of the (P.), 28
Small-pox: Epidemic at Dewsbury, The, 62; and Vaccination (P.), 226
Smoke and Fog (A.), 169
Society of Medicine, The Proposed Royal, 418
Sociology, Modern, 302
Sodium Chloride, Excretion of (P.), 408
Spine: Cervical, Extensive Fracture, Dislocation of the, Producing no Symptoms (P.), 283; Fracture of, Complicated by Large Prostatic Calculus (P.), 282; Injuries to Cord and Column (P.), 282; Spinal Cord, Arterio-sclerosis of the (P.), 465; Spinal Cord, Laminectomy for Injuries of the (P.), 27; Acute Osteomyelitis of the (P.), 28; Acute Perimeningitis (P.), 28
Spleen, Primary Sarcoma of the (P.), 352
Sponges, Surgical, 371
Statistics: Anti-vivisection, 12, 17; Criminal (A.), 24; London, Some (A.), 37; Public Health, the Organisation of (A.), 327
Stenosis of the Pylorus, Congenital Hypertrophic (C), 44, 463
Sterility due to the Male (P), 71
Still-born Children, Rigor Mortis in (C.), 67
Stimulants and Human Progress, Nervine (A.), 3
Stomach: Chronic Disorders (P.), 89; Diseases of the, Diagnostic Methods in (P.), 70; Diseases of the (P.), 88; Hour-glass (P.), 266; and Intestines, Diseases of the (P.), 190, 209; Malignant Disease (P.), 89; Progress in Surgery of the (P.), 246, 266; Tetany due to Foreign Bodies in the (C.), 315
Streptothricosis (P.), 466
Streptothrix Infection (P.), 27
Strychnine as an Evacuant (C.), 8
Subscriptions and Medical Schools, Hospital, 379
Surgery: Bloodless (A.), 293; Gross Cleanliness in (C.), 106; Surgery (C.), 245; Appendix, Vermiform (P.), 332; Bone (P.), 143; Cerebral and Nerve (P.), 177; Cerebral, Spinal, and Nerve (P.), 27; Cerebral (P.), 282; Genito-Urinary (P.), 227, 249, 368, 389; Hernia (P.), 264, 283, 447; Intestines (P.), 425; Liver, 351; Pancreas and Spleen (P.), 352; Peritoneum (P.), 248; Sarcoma of the Spleen, Primary (P.), 352; Stomach (P.), 246, 266
Surgical Aid Society, the (W.), 197
Synovitis in Children, Syphilitic (C.), 139

Syphilis (C.), 44; Inherited (C.), 44; Intracranial (P.), 175; of Upper Air Passages (P.), 90. *See* Book World, *s.v.* Lambkin; The Treatment of (P.), 389

Tabes Dorsalis (C.), 42; and Disseminated Sclerosis (C.), 120; The Pains of (C.), 404
Tattooing and Disease (A.), 169
Teachers and Examiners (A.), 419
Teeth, Neglect of, 38
Tetanus, The Treatment of, by Antitoxin (P.), 177
Tetany: due to Foreign Bodies in the Stomach (C.), 315; Gastric Dilatation and (P.), 266
Therapeutical Society, The (A.), 185
Throat. *See* Book World, *s.v.* Lamb; Disease at Lincoln (A.), 21; Organism, a New Pathogenic (P.), 226
Thrombosis of the Cavernous Sinus (P.), 8; and Embolism of the Vessels of the Mesentery (P), 426
Thymus, The (P.), 350
Thyroid Gland, Internal, Secretion and the (C.), 156
Thyroid and Parathyroids, The Function of the (C.), 463
Tic, Convulsive (C.), 462
Tissue-lymph Circulation (P.), 316
Titles, The Question of (A.), 277
Toilette in the Seventeenth Century, Medical Directions for a, 336
Tongue, Epithelioma of the, in Women (C.), 106
Toxicology. *See* Book World, *s.v.* Brundage
Toxines. *See* Book World, *s.v.* Bosanquet
Tracheotomy and Sutubation (C.), 298
Tramway Servants, Seats for (A.), 135
Transplantation of Tendon for Musculo-spiral Paralysis (P.), 177
Trauma in Relation to Disease of the Nervous System (P.), 125
Treatment, The Neglect of Medical (A.), 83
Tropical Medicine, Examinations in, 150
Trustees and their Obligations (A.), 259
Trypanosoma from the Leishman-Donovan Body, The Cultivation of (C.), 281
Trypanosomiasis in the Congo (C.), 47
Tuberculin, The Us eof (C.), 45; as a Diagnostic Agent (P.), 370
Tuberculosis (C.), 243, 370, 448; The Conference on, 12; Marriage and (A.), 3; and other Diseases (P.), 51; Treatment of (P.), 427. *See* Book World, *s.v.* Burton-Fanning; Bovine (P.), 466; The Early Diagnosis of (P.), 427; Feeding in (P.), 465; and Heart Disease (P.), 449; The Treatment of Laryngeal (C.), 349; Urinary (P.), 227
Tumour, Cerebral, Hemiplegia in (P.), 464; of the Brain (P.), 28; Cervical Intradural, Removal of a (P.), 282; Intracranial (C.), 40; of the Pituitary Body (P.), 9
Tumours and Insanity: Frontal Lobe (P.), 299 involving the Optic Chiasma (P.), 282

Ulcer: Duodenal, and its Treatment (C.), 172; Gastric (P.), 88, 246; Gastric, Bacteriology of (C.), 189; Gastric, Orthoform in the Diagnosis of (C.), 88; Gastric, Parotitis 207; Paralysis of the Palmar Branch of the (P.), 300; Rodent (C.), 281
Uncinariasis (P.), 90
Unemployed, The Mansion House Committee and the (A), 311
Ureter, the Treatment of Accidental Wound of the (P.), 107
Urethra, Bulbous, Carcinoma of the (P.), 71; Repair of the, by Transplantation of the Urethra of Animals (P.), 368; The Treatment of Ruptured (P.), 369
Urethritis, Kollman Five-glass Test in (P.), 106
Urine, Chemical Examination of the (P.), 126; Examination. *See* Book World, *s.v.* Carruthers.
Urines, Intravesical Separation of the (C.), 446
Uterus, Hernia of the, through the Inguinal Canal (P.), 285; Inversion of the (P.), 210; Retroversion of the Gravid (P.), 210

Vaccines. *See* Book World, *s.v.* Bosanquet.
Vaccination in Canada, 260
Vagrants and Infectious Disease, 118, 134
Varicose Veins (C.), 205
Ventilation of Railway Carriages, 64
Visit of French Medical Men to London, The (W.), 59, 65, (W.), 271, 307
Victoria, Illness of Princess (A.), 327
Vomiting, Anæmic (C.), 47; of Enemata (C.), 7
Vote, Admission by, a Practical Protest against (A.), 381

Water Softener (W.), 199
Waters, Aërated, 310
Whitlow, Acute Diphtheritic (C.), 222
Women, Diseases of. *See* Book World, *s.v.* Bland-Sutton and Macnaughton Jones
Word-Blindness, Congenital (P.), 465
Wounds in War. *See* Book World, *s.v.* Stevenson

*X*-rays as an Aid to Medical Diagnosis (C.), 104; *See* Book World, *s.v.* Cowen

### THE SPECIAL SECTION FOR NURSES.

Acland Homes, The, 329
Age Question, The Local Government Board and the, 79
Aged, Nursing the, 17
Agreements, The Value of Written, 329
Albany, Duchess of, at the Evelina Hospital, 105; at the National Hospital for the Paralysed, 41
Anglo-American Nursing Home at Rome, The, 296
Appointments, 10, 22, 44, 58, 72, 86, 98, 112, 126, 138, 159, 171, 182, 196, 212, 224, 237, 250, 262, 273, 286, 298, 312, 322, 338, 350
Appointments by Poor-Law Guardians, 91
Association for Promoting the Training and Supply of Midwives, 284
Associations, Nursing Guardians and Contributions, 255

Bakewell Workhouse Infirmary, Scandal at, 305
Balfour, Presentation to Miss, 241
"Benefits for Trained Nurses," 169
Berlin, English Nursing in, 89
Beyond the Seas: Nursing in the Australian Bush, 7, 20; Cape Town, Somerset Hospital, 234; Foreign Nurses in Japan, 55; Nursing in the Russian-Dutch Field Hospital at Taolaitschao, 83, 195; District Nursing in the South Sea Islands, 31
Book and its Story, A, 63, 75, 115, 185, 198, 239, 275, 301, 327
Books on Nursing, New, 46, 111
Books for Nurses, New, 299
Boston Floating Hospital, The, 323, 347
Brisbane Hospital, Post-Graduate Certificates for, 163
Brompton Cancer Hospital, Nurses' Home at the, 71, 105
Bury, Paying Patients at, 189
Buxton, Devonshire Hospital, The King and Queen at, 215

California State Nurses' Association, The, 2
Capetown, Victoria Institute, 89
Certificate, The Value of the Croydon, Restored, 158, 175
Certificates, 29, 73
Charing Cross Hospital, The Nurses of, 309
Cheltenham General Hospital, The Nurses of the, 181
Children, Crippled, The Nursing of, 15
Chiropody and Nursing, 4
Christian, Princess, and the Nurses of the Somerset Hospital, Capetown, 77
Christian, Princess, at Pretoria, 49
Christmas Books, 160, 173, 183
Christmas: in the Hospitals, 193, 209; in the Fever Hospitals, 145
City of London Lying-in Hospital, the Pupils at the, 348
Clapton, Opening of a Nurses' Settlement at, 169
*Class Legislation*, 5, 43, 73, 86, 100, 212
Common Sense in Nursing Affairs, 269
Cookery Examinations at Camberwell, 4
Corea, Progress of Nursing in, 215
Croydon Infirmary and Probationers, 97
Crumpsall Infirmary, 79
Cubicles, the Case against, 201, 215, 217, 224
Curzon's, Lord, Gratitude, 165

Damages for a Nurse, Substantial, 331
Dance at Liverpool, Nurses', 277
Day in a Dispensary, a, 235
Death in our Ranks, 83, 250, 286, 322
Devonshire, a County Association for, 79
Difficulties, Practical Nursing, 345
Dismissal for Disobedience, 51
Dublin: "Margaret Huxley" Prize, 89; Protestant Hospital, 253

East London Nursing Society, 171
East Preston Guardians, the Untrained Matron and the, 119
Echoes from the Outside World, 11, 23, 47, 62, 74, 87, 101, 114, 127, 139, 172, 184, 199, 213, 225, 238, 251, 263, 274, 287, 300, 313, 326, 339, 352
Emigration of Poor-law Children, the, 245
Everybody's Opinion, 9, 22, 43, 61, 72, 86, 99, 113, 126, 138, 159, 170, 182, 197, 212, 224, 236, 249, 262, 273, 286, 299, 325, 337, 351
Examination Nightmare, an, 324
Examination Questions for Nurses, 9, 34, 110, 135, 350

Fees, Guardians' and District, 187
Fever, Scarlet, Infection, 84, 110

Glasgow: "Alice Mary Corbett" Nurses' Home; 91; the New Hospitals at, 2
Grimsby, the Nurses' Home at, 227
Guy's Hospital Trained Nurses' Institution, 49

Help the Nurses to help themselves, 171, 183
Hints, Practical: Coffee, 351
Holidays, District Nurses, 182
Honours for a Scottish Nurse, 129
Hospital, The, Convalescent Fund, 183
Hospital for Mothers and Babies, 217

Incorporated Society for Promoting Higher Education and Training of Nurses, The, 281, 319, 329, 345

India : Home for Nurses in, 201 ; The Originator of the Nursing Service in, 201
Infant Life Protection in South Australia, 321
Infirmaries, Poor Law, Pauper Attendants in Lancashire, 317
Irrigation to an Injured Leg, the arrangement of Constant Hot, 283

Journal for Canadian Nurses, A, 332
Junius E. Morgan Benevolent Fund, The, 183

Kaiserswerth, Nursing at, 35
King and Queen at Devonshire Hospital, Buxton, 215

Lectures at Guy's, Post Graduate, 25
Lectures upon the Nursing of Infectious Diseases : Chicken Pox, 320 ; Croup, Diphtheritic, 168, 192 ; Diphtheria, 30, 70 ; The Antitoxin Treatment of, 94 ; Fever, Enteric, 346 ; Fever, Scarlet, 6 ; Measles, 220 ; Measles, German, 246 ; Paralysis, Diphtheritic, 122 ; Small-pox, 270, 294, 320
Lectures upon the nursing of Mental Diseases, 18, 54, 82, 108, 134, 180, 206, 232, 258, 282
Lectures on the Nursing of Sick Children, 308, 334
Lepers of the Transvaal, 38
Lincoln Typhoid Epidemic, The Nursing Arrangements, 311
Liverpool, The Nurses of the David Lewis Northern Hospital, 33
London, Bible Women and Nurses' Mission, 92
London Hospital, Nursing Jewish Patients at the, 105

Madras, The New Institute in, 65
Managers, Women as Hospital, 133, 159, 170, 182, 197, 224, 236, 249, 257
Manchuria, Scarcity of Nurses in, 341
Massage at the City of London Chest Hospital, 28 ; The Treatment of, 15
Maternity, Training at Belfast, 243
"Medical Register" Swindle, the, 71
Mental Cases, The Nursing of, 107
Midwife : Levelling up the, 179 ; The Passing of the Incompetent, 241
Midwifery, District Nursing and, 289, 325 ; Training at the East End Mothers' Home, 256
Midwives, Poor-law Infirmaries and the Training of, 215 ; The Salaries of, 113 ; The Training of, 95 ; Women Inspectors of, 227, 236, 249, 255, 285, 299, 325, 338 ; Work of the, 307
Midwives Act, The, 4 ; Manchester Corporation and the, 201 ; Poor-law Infirmary Nurses and the, 305, 331
Midwives Board, Central, 21, 85, 137, 189, 248, 261, 284, 298, 312, 349 ; The Examinations of the, 255 ; The Functions of the, 13 ; and Poor-Law Infirmaries, 143
Milk Supply, Nurses and the, 93
Misconduct at a County Asylum, 189
Mortality in Russia, Infantile, 271

Nightingale, Florence : The Life of, 59 ; The Jubilee of, 53, 60 ; and the South Australian Nurses, 105 ; and South London Nurses, 163
Northallerton, A Retrograde Proposal at, 131
North Devon Infirmary, A New Departure at, 131
Northwood Hospital for Consumptives, 1
Norwich Union Nurses' Home, 27
Notes on News from the Nursing World, 1, 13, 25, 49, 65, 77, 89, 105, 117, 129, 141, 163, 175, 189, 201, 215, 227, 241, 253, 265, 277, 289, 303, 315, 329, 341
Notes and Queries, 12, 24, 48, 64, 76, 88, 116, 128, 140, 162, 174, 186, 200, 214, 226, 240, 252, 264, 276, 288, 302, 314, 328, 340, 354
Novelties for Nurses, 45, 100, 137, 250, 298, 350
Nurse Tried for Killing a Patient, An Asylum, 267
Nurse in Tuberculous Cases, The Visiting, 91
Nurseries, The New Norland, 141, 157
Nurses Beware ! 293
Nurses Co-operation, The, 277
Nurses in Court, 261
Nurses, The Japanese Army, 158
Nurse's Limit, The, 121, 133
Nurses in Lodgings, Poor Law Infirmary, 277
Nurses for the Middle Classes, 42, 69, 86, 100, 113
Nurses Missionary Union, 52
Nurses and Touts, 119
Nursing Association, Colonial, 171
Nursing, District, and Trades Councils, 203
Nursing in the Russian-Dutch Field Hospital at Taolaitschao, 195
Nursing, The Standard of Private, 93
Nursing the Victims of the Cudworth Railway Accident, 244

Obstetrical Society of London, The, 318
Obstetrics in a Caravan, 40
Opening for Elderly Nurses, A New, 229
Outlook, The Nursing, 5, 17, 29, 53, 69, 81, 93, 107, 121, 133, 144, 167, 179, 191, 205, 219, 231, 245, 257, 269, 281, 293, 307, 319, 333, 341

Paris, Nursing at the Pasteur Hospital, 56
Paris Probationers, The Condition of the, 2
Pensions for Glasgow Nurses, 163 ; at the Middlesex Hospital, Nurses, 136
Poplar and Stepney Sick Asylum, The Nurses of the, 123
Presentation Craze, The, 99
Presentation, 71, 112, 138, 183, 272, 236, 310, 325
Probationers, The Appointment of Poor Law, 189

Queen Alexandra and the Queen's Nurses, 117
Queen Alexandra's Imperial Military Nursing Service, 291, 315, 343
Queen's Nurses : in Elementary Schools, 341 ; and the Holt-Ockley System, 267 ; Increasing Demands for, 253 ; the New, 241 ; and the Poor Rate, 331 ; Scottish, and Edinburgh Infirmary, 178
Queen Victoria's Jubilee Institute for Nurses, 171, 247

Reading to the Sick, For, 12, 24, 48, 64, 76, 88, 102, 116, 128, 140, 162, 174, 186, 200, 214, 226, 240, 252, 264, 276, 288, 302, 314, 328, 340, 354
Registration Bill, Fate of the Nurses', 315
Registration : Miss Monk on, 219 ; State, 230, 249, 281, 297, 303, 315, 329, 345, 351 ; State, and Poor-Law Matrons, 126 ; Voluntary and Compulsory, 197
Resignation of a Matron, 67
Royal Free Hospital, the New Matron of the, 289
Royal National Pension Fund for Nurses, 167, 171, 333, 335
Rural Midwives Association, 256

St. Mary's Hospital, A Royal Surprise Visit, 329
Salisbury Home, Personal Experiences in a, 109
Sanitation, Nurses and, 205
Savings, A Nurse's, 167
School Nurse, The, 191
Self-help and Co-operation, 333
Sick Pay, Nurses and, 231
Sick Room Help Society, 168
Sponging a Fever Patient with Tepid Water, 221
Stafford Union Infirmary, An Improvement at, 241
Stage Army "At Home," A, 297
Status of the English Nurse, 65
Sunderland, Ryhope Asylum, Ultimatum of Nurses, 277, 299
Sunderland Workmen and District Nursing, 279
Sussex County Hospital, The Nurses of, 259
Sutherland Benefit Nursing Association, The, 119

Testimonials by Guardians' Committees, 325
Tests, Religious, Southwark Guardians and, 129
Training : at a Children's Hospital, Excessive, 73 ; Educated Women to be Nurses in Paris, 233 ; at Fever Hospitals, 315 ; of Mental Nurses on Hospital Lines in Theory and Practice, 207, 223 ; at a Nursing Institute, 311, 325 ; Quality of, 10 ; School at the General Lying-In Hospital, Reorganisation of the, 285
Trained Nurses' Annuity Fund, 317, 338, 351
Travel Notes and Queries, 22, 44, 60, 71, 83, 99, 136, 197, 322, 338, 351
Truro, a Wise Decision at, 189
Twining, Miss Louisa, 125
Typhus Epidemic, Nursing in a, 295

Ulcer, How to Strap a Leg for the Cure of an, 135

Valencia Village Hospital, Ireland, A Visit to the, 61
Victorian Order of Nurses, The, 331
Visit to a Japanese Nursing Home, A, 222

War Time, Nurses for, 144
Wedgwood, The Resignation of Miss, 227
West London Hospital, Farewell of the Lady Superintendent, 211
Winter Abroad, 81
Workhouse Nursing, The Development of, 57

Printed by SPOTTISWOODE & CO. LTD., New Street Square, E.C. ; and Published by the Proprietors, THE SCIENTIFIC PRESS, LIMITED, at 28 & 29 Southampton Street, Strand, London, W.C.

### NOTICE TO CORRESPONDENTS.

All MSS., Letters, Books for Review, and other matters intended for the Editor should be addressed The Editor, "The Hospital," The Hospital Buildings, 28 & 29 Southampton Street, Strand, W.C.

The Editor cannot undertake to return rejected MSS., even when accompanied by stamped directed envelope.

**Notice to Advertisers and Subscribers.**

All Advertisements, Orders for Copies of The Hospital, and Business Communications should be addressed to **THE MANAGER (Not to the Editor),** The Hospital, 28 & 29 Southampton Street, Strand, London, W.C.

The Cost of Subscription, post free, is as follows:—Per week, 3½d.; per quarter, 3s. 3d.; per half-year, 6s. 6d.; per annum, 13s. Foreign and Colonial:—Per annum, 19s. 6d., payable, in all cases, in advance.

**NOTICE.—Vol. XXXV. of "The Hospital" (October 1903 to March 1904), in handsome cloth binding, is now on sale at the office of this Journal, price 10s. 6d. Cases for binding the Numbers contained in this Volume 2s. Index to Vol. XXXV., 3½d., post free.**

**The Monthly part for October is now ready. Price 1s., by post, 1s. 3d.**

### NOTES AND NEWS.

### THE HOSPITAL LIBRARY AND CHARITIES BUREAU.

Inquiries of the librarian are answered in this column free of charge. Inquiries to be answered promptly by post must be accompanied by a fee of 2s. 6d.

The Librarian announces the addition of 24 volumes of the Encyclopædia to the Library for the use of members.

Hospital Manager asks: Can securities be held in the name of the institution to obviate the difficulties arising when securities are held in the names of individual trustees?

It would seem best to invest the funds in the names of the permanent officials, or to place them with the Charity Commissioners to act as trustees. The Charity Commissioners would act only as requested by the institution and do not interfere in administration.

### Medical Appointments.

T. H. Bailey, L.S.A., District Medical Officer of the Nantwich Union. **Edward Percy Calder**, M.B., Ch.B.Edin., Resident Medical Officer to the Royal Lancaster Infirmary. **R. Catto**, M.B., Ch.B.Aberd., House Surgeon, Aberdeen Royal Infirmary. **A. Dyce Davidson**, M.A., M.B., Ch.B.Aberd., House Physician, Aberdeen Royal Infirmary. **Patrick J. King**, M.B., Ch.B.Glasg., Assistant Surgeon to the Dispensary of the Royal Hospital for Sick Children, Glasgow. **J. U. MacMullan**, L.R.C.P.&S.Edin., L.F.P.S.Glasg., Surgeon and Agent to the Admiralty at Penarth. **P. R. Tarbet**, M.R.C.S., L.R.C.P., Medical Officer of Health, Stevenage Urban District. **S. W. Thompson**, M.R.C.S., L.S.A., Medical Officer, Calvert Road Homes, Greenwich Union. **A. B. Timms**, L.R.C.P.&S.Edin., Assistant Medical Officer of the Cardiff Workhouse. **G. Benington Wood**, M.B., C.M.Edin., Medical Officer to the Post Office and Medical Examiner to the Board of Education, Sandown, Isle of Wight.

Edinburgh Royal Infirmary.—The following appointments were confirmed by the Board on September 12th: Clinical Tutors for the ensuing winter and summer sessions, 1904-1905.—**G. F. Barbour Simpson**, M.B., F.R.C.S.Edin. (Gynæcological Department); **Edwin Matthew**, M.A., M.B., Ch.B.; **George Lyon**, M.B., Ch.B.; and **Edwin Bramwell**, M.B., M.R.C.P.Edin.& Lond., University Medical Wards; **M. Herbert Shepherd**, M.D., F.R.C.S.Edin., University Surgical Wards. Medical Wards: **Alexander Goodall**, M.D., F.R.C.S. (Dr. James); **J. D. Comrie**, M.A., M.B., Ch.B. (Dr. Bramwell); **W. T. Ritchie**, M.D., M.R.C.P.Edin. (Dr. Gibson); **W. B. Drummond**, M.B., C.M (Dr. Bruce). Surgical Wards: **H. T. Thomson**, M.D. (Mr. Cotterill); **A. Campbell Geddes**, M.B., Ch.B. (Mr. Cathcart). Instructors in Anæsthesia: **T. T. Luke**, F.R.C.S.Eng., to Professor Annandale's wards; **G. W. B. Daniel**, M.R.C.S., L.R.C.P., to Mr. Cathcart's wards. Resident Physicians: **G. Stirling Landon**, M.B., Ch.B., to Professor Greenfield; **Hugh S. Davidson**, M.B., Ch.B., to Dr. Bramwell; **R. W. Johnstone**, M.A., M.B., Ch.B., to Dr. Gibson; **J. Allen Anderson**, M.B., Ch.B., to Dr. Bruce. Resident Surgeons: **T. P. Caverhill**, M.B., Ch.B., to Professor Annandale; **D. Brown**, M.B., Ch.B., to Dr. Berry Hart; **Henry Curwen**, M.B., Ch.B., to Mr. Cotterill; **A. G. Coullie**, M.B., Ch.B., to Mr. Cathcart. Clinical Assistants: **James M. Graham**, M.B., Ch.B., to Dr. Bramwell; **D. P. D. Wilkie**, M.B., Ch.B., to Dr. Bruce; **Thomas E. Coulson**, M.B., Ch.B., to Dr. MacGillivray; **W. A. Wilson Smith**, M.B., Ch.B., to Mr. Cathcart; **Arthur J. Brock**, M.B., Ch.B., to Dr. Murdoch Brown in the medical waiting room.

### "The Hospital" Institutional Library.

**"Hospitals and Asylums of the World." 4 Vols., with a Portfolio of Plans, complete £12 12s.**

**"Burdett's Hospitals and Charities, 1904." 5s. net.**

**"Burdett's Official Nursing Directory." 3s. net.**

**"Cottage Hospitals, General, Fever, and Convalescent." 10s. 6d.**

**"Uniform System of Accounts for Hospitals and Public Institutions." New Edition. 4s. net.**

**"Hospital Expenditure: The Commissariat." 2s. 6d.**

**"A Legal Handbook for the Use of Hospital Authorities." 2s. 6d.**

**"The Cottage Hospital Case Book or Register of Patients." 10s. and 12s. 6d.**

**All these are published by the Scientific Press, Ltd., and may be obtained through any bookseller, or direct from the publishers, 28 and 29 Southampton Street, Strand, London, W.C.**

### Official and Medical Appointments.

AN EXAMINATION OF CANDIDATES for entry into the Medical Department of the Royal Navy, will be held in November next at Examination Hall, Thames Embankment.

The forms to be filled up by Candidates will be supplied on application to the Medical Director-General,

Admiralty,

18 Victoria Street, S.W.

1st September, 1904. (53)

SEAMEN'S HOSPITAL SOCIETY, GREENWICH, S.E.

The Committee invite Candidates for the following appointments:—

JUNIOR RESIDENT MEDICAL OFFICER, Dreadnought Hospital, Greenwich. Salary £40 per annum, with Board, Residence, and Washing.

HOUSE SURGEON, Branch Hospital, Royal Victoria and Albert Docks, E. (which is in connection with the London School of Tropical Medicine). Salary £50 per annnm, with Board, Residence, and Washing.

Candidates must be doubly qualified and registered. Applications, stating age, together with copies of not more than three recent testimonials, to be sent in, on or before 4th of October, to the undersigned from whom further particulars may be obtained.

By Order,
P. MICHELLI, Secretary.

Greenwich, September 13, 1904. (65)

COUNTY BOROUGH OF HANLEY.

(Area 1,763 acres. Population 63,000.)

WANTED, LADY ASSISTANT MEDICAL OFFICER to act as supervising officer under the Midwives Act, and as a Sanitary Inspector.

Applications, stating age, qualifications, and salary required, to be accompanied with copies of not more than three recent testimonials, to be forwarded to me on or before TUESDAY, October 4th, 1904.

The lady appointed will be required to devote the whole of her time to the duties.

ARTHUR CHALLINOR, Town Clerk.

Town Hall, Hanley. (88)

### The Modern Nurses' Library.

| | | |
|---|---|---|
| Thoroughly up to Date. | **A HANDBOOK FOR NURSES.**<br>By J. K. WATSON, M.D., M.B., C.M.<br>New and Revised Edition. | 5/- net,<br>5/4<br>post free. |
| With List of Instruments. Profusely Illustrated. | **SURGICAL WARD-WORK AND NURSING.**<br>By ALEXANDER MILES, M.D.Edin., C.M., F.R.C.S.E. | 3/6 net,<br>3/10<br>post free. |
| A comprehensive Guide for Eye Cases. | **OPHTHALMIC NURSING.**<br>By SYDNEY STEPHENSON, M.B., F.R.C.S.E.<br>With a list of Instruments required, and a Glossary of Terms. | 3/6 net,<br>3/10<br>post free. |
| Essential for Special Cases. | **NURSING IN DISEASES OF THE THROAT, NOSE, AND EAR.**<br>By P. MACLEOD YEARSLEY, F.R.C.S. Illustrated. | 2/6<br>post free. |
| A simple and able Manual. | **GYNÆCOLOGICAL NURSING.**<br>By G. A. HAWKINS-AMBLER, F.R.C.S. | 1/-<br>post free. |
| Very practical. | **FEVERS & INFECTIOUS DISEASES:**<br>**Their Nursing and Practical Management.**<br>By WILLIAM HARDING, M.D. | 1/-<br>post free. |
| A good introduction to the subject. | **NOTES ON PHARMACY AND DISPENSING FOR NURSES.**<br>By C. J. S. THOMPSON. New and Revised Edition. | 1/-<br>post free. |

**The Scientific Press, Ltd.,**
28 & 29 Southampton St., Strand, London, W.C.

*Publishers and Booksellers of every kind of Nursing Text-Books, Case-Books and Charts.*

**SEND FOR NEW CATALOGUE.**

